# The Relationship between Frontal Lobe Lesions, Course of Post-Stroke Depression, and 1-year Prognosis in Patients with First-Ever Ischemic Stroke

**DOI:** 10.1371/journal.pone.0100456

**Published:** 2014-07-08

**Authors:** Yu-Zhi Shi, Yu-Tao Xiang, Shuo-Lin Wu, Ning Zhang, Juan Zhou, Ying Bai, Shuo Wang, Yi-Long Wang, Xing-Quan Zhao, Gabor S. Ungvari, Helen F. K. Chiu, Yong-Jun Wang, Chun-Xue Wang

**Affiliations:** 1 Department of Neurology, Beijing Tian Tan Hospital, Capital Medical University, Beijing, China; 2 China National Clinical Research Center for Neurological Diseases, Beijing, China; 3 Center of Stroke, Beijing Institute for Brain Disorders, Beijing, China; 4 Beijing Key Laboratory of Translational Medicine for Cerebrovascular Disease, Beijing, China; 5 Faculty of Health Sciences, University of Macau, Macao SAR, China; 6 Department of Psychiatry, Chinese University of Hong Kong, Hong Kong SAR, China; 7 Mood Disorders Center, Beijing Anding Hospital, Capital Medical University, Beijing, China; 8 Department of Neurology, Beijing Daxing District Hospital, Capital Medical University, Beijing, China; 9 Xinjiang Production and Construction Corps 13 division Red Star Hospital, Xinjiang, China; 10 School of Psychiatry and Clinical Neurosciences, University of Western Australia, Perth, Australia; 11 University of Notre Dame Australia/Marian Centre, Perth, Australia; University of Muenster, Germany

## Abstract

**Background and Purpose:**

Most studies on post-stroke depression (PSD) have focused on a certain time point after stroke instead of the time course of PSD. The aim of this study was to determine the relationship between frontal lobe lesions, course of PSD over a year following the stroke onset, and the 1-year prognosis in patients with first-ever ischemic stroke.

**Methods:**

A total of 1067 patients from the prospective cohort study on the incidence and outcome of patients with post stroke depression in China who were diagnosed with first-ever ischemic stroke and attended 4 follow-up visits at 14±2 days, 3 months, 6 months, and 1 year after stroke onset, were enrolled in the study. PSD was diagnosed according to DSM-IV. The course of PSD was divided into the following two categories: persistent/recurrent depression and no/transient depression. Patients with any ischemic lesion responsible for the indexed stroke event located in the frontal lobe were defined as patients with frontal lobe lesions. Modified Rankin Scale (mRS) ≥2 at 1-year was considered to be poor prognosis.

**Results:**

There were 109 patients with and 958 patients without frontal lobe lesions that formed the frontal lobe (FL) and no-frontal lobe (NFL) groups, respectively. After adjusting for confounding variables, frontal lobe lesion was significantly associated with persistent/recurrent PSD (OR 2.025, 95%CI 1.039–3.949). Overall, 32.7% of patients in the FL group had poor prognosis at 1- year compared with 22.7% in the NFL group (*P* = 0.021). Compared with no/transient depression, persistent/recurrent depression was found to be an independent predictor of poor prognosis at 1-year both in FL and NFL groups.

**Conclusions:**

Long-term and periodical screening, evaluation and treatment are needed for PSD after the onset of ischemic stroke, particularly for patients with frontal lobe infarction.

## Introduction

Post-stroke depression (PSD) generally has a chronic course and contributes to a variety of adverse health outcomes, including increased disability, morbidity and mortality [1], [2]. A systematic review of observational studies indicated that depressive symptoms were present in approximately one third of all stroke survivors at any time during their follow-up [3]. The prospective cohort study on the incidence and outcome of patients with PSD in China (PRIOD) reported that the 1-year cumulative incidence of PSD was as high as 41.8% [4].

Whether the locations of stroke lesions are related to PSD have been researched for decades, but the question remains unanswered. Previous studies have demonstrated morphological changes in the frontal lobe (left, right or bilateral) [5]–[7] and left basal ganglia [8] in patients with PSD, and some studies have suggested that the anatomical correlation was dynamic and changed over time [9]. Other studies have reported no correlation between PSD and lesion locations [10]. The lack of consensus about the relationship between lesion locations and PSD might be due to different study methodology, including the source of study populations, timing of first interview after stroke, and measurement of depression [11]. A number of recent studies have demonstrated that patients with lesions in the frontal lobe had a higher risk of PSD and have provided some pathophysiological underpinnings for PSD [12], [13]. Whether frontal lobe lesions are associated with the course of PSD is still unknown.

The PRIOD study found a definite relationship between the course of PSD and 1-year unfavorable prognosis in patients with ischemic or hemorrhagic stroke and showed that persistent, recurrent and transient PSD over a year after stroke onset were significantly associated with a higher risk of functional dependence (mRS≥2) at 1-year. Among the different time courses of PSD, persistent depression and recurrent depression had higher predictive values than transient depression [4].

Based on the information on the relationship between frontal lobe lesions and PSD, and the association of the course of PSD and functional outcome, we hypothesized that in patients with first ischemic stroke: (1) frontal lobe lesions, detected by common MRI sequences (T1 and T2 weighted, fluid-attenuated inversion-recovery sequence, diffusion weighted imaging) or CT scans, would be associated with persistent/recurrent course of PSD; (2) patients with frontal lobe lesions would have worse prognosis than patients with ischemic lesions located elsewhere. To test these hypotheses, the baseline and 1-year follow up data of the PRIOD study were re-analyzed.

## Methods

### Study participants and settings

This study re-analyzed data from the Prospective cohort study on the Incidence and Outcome of patients with post stroke Depression in China (PRIOD). Details of the PRIOD project have been described elsewhere [4]. Briefly, the inclusion criteria for the PRIOD study were: age ≥18 years; fulfilling the definition of stroke according to WHO criteria and confirmed by CT or MRI; onset of stroke within 14 days of admission; consent to participate in the study signed by the patient or a legally authorized representative. Exclusion criteria were: previous history of dementia or other known neurological diseases that could affect cognitive functions; history of alcohol or drug abuse; and inability to communicate appropriately, including aphasia. All patients enrolled in the PRIOD study were expected to accomplish 5 visits at baseline, 14±2 days, 3 months, 6 months, and 1 year, respectively.

**Figure 1:**
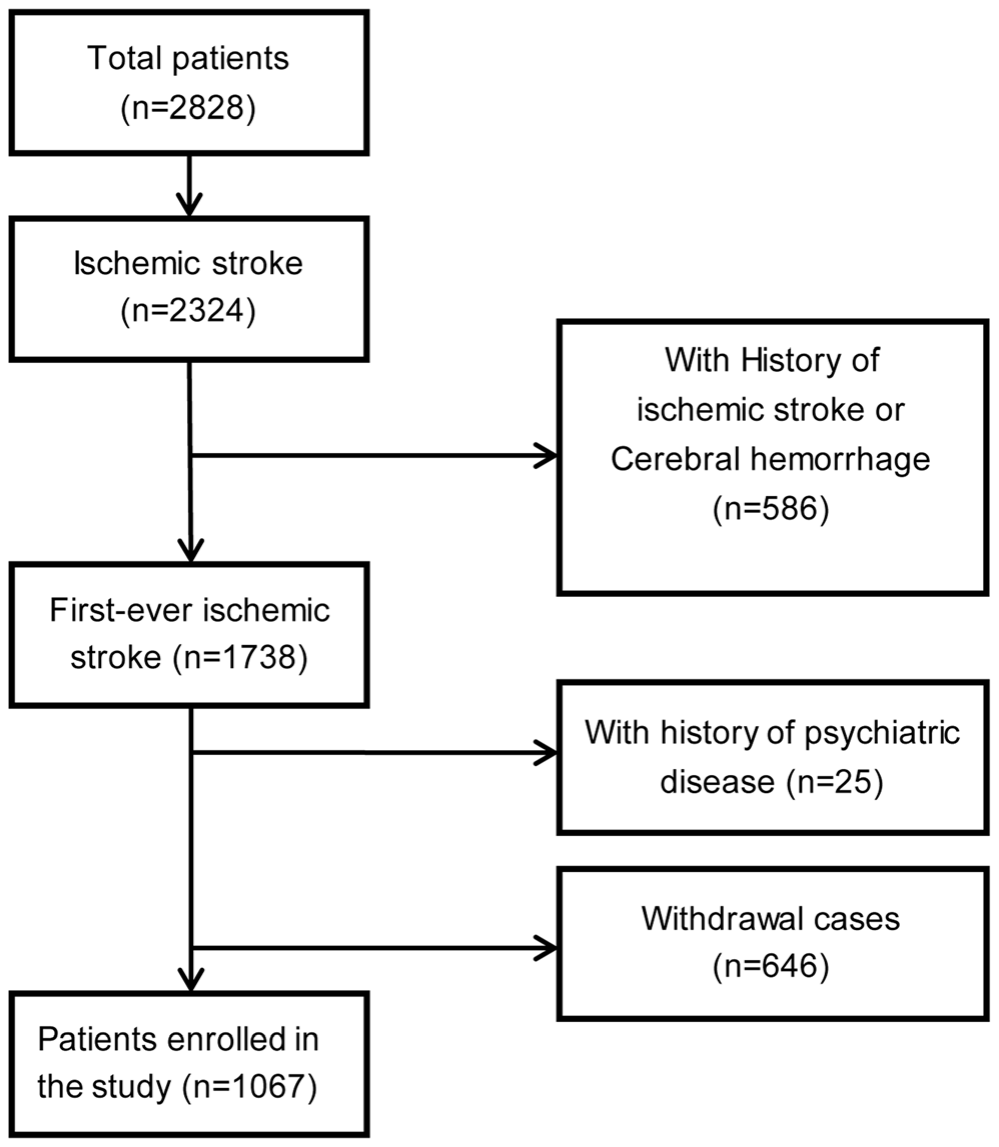
Patients' inclusion chart.

A total of 2,828 patients with stroke were recruited from neurology departments of 56 hospitals nationwide in China between April 2008 and April 2010. Patients were excluded from the present analyses if they were diagnosed with cerebral hemorrhage or subarachnoid hemorrhage; had a history of stroke or psychiatric disease (depressive disorders, anxiety disorders and major psychiatric comorbidity); or withdrew from follow-up at any of the following time points: 14±2 days, 3 months, 6 months and 1 year. At last, 1,067 patients fulfilled these inclusion and exclusion criteria and were included in the study (Figure 1).

The PRIOD study was approved by the Medical Ethics Committee of Beijing Tian Tan Hospital, Capital Medical University. The study was conducted in compliance with the Declaration of Helsinki Guidelines for Protection of Human Subjects. All participants provided written consent.

### Study instruments and assessments

Basic demographic data, medical history, and habits of smoking and drinking were collected. The National Institutes of Health Stroke Scale (NIHSS) and the Mini Mental State Examination (MMSE) were administered on admission to evaluate the severity of stroke and cognitive impairment. Details of antidepressant treatment were recorded at each time point of follow-up.

The locations of ischemic lesions responsible for the indexed stroke event on MRI and CT scans were identified and reported by radiologists of each study center who were blinded to the patients' psychiatric diagnoses. Of the 1,067 patients enrolled in the analyses, 881(82.6%) had a MRI scan and the rest had a CT scan. Patients with any ischemic lesion located in the frontal lobe were assigned to the frontal lobe (FL) group, while those without frontal lobe lesions were assigned to the no-frontal lobe (NFL) group.

Diagnosis of depression was performed by well-trained physicians according to the Diagnostic and Statistical Manual of Mental Disorders, fourth edition (DSM-IV) [14]. The Hamilton Rating Scale for Depression-17 (HRSD-17) was used to assess the patients' depression severity degree [15]. The PRIOD study used the Chinese version of the HRSD-17. HRSD-17 was administered at all 4 follow-ups. Raters had received standard training and were blinded to the patients' clinical information.

Persistent depression was defined as having a diagnosis of depression at least 2 consecutive follow-up points and the depression symptoms were persistent since the diagnosis of depression. Recurrent depression was defined as being diagnosed with depression at non-consecutive follow-up points. Transient depression was defined as depression symptoms that had completely relieved at subsequent follow-up point after diagnosis and without recurrence [4], [16]. Patients enrolled in the analyses were divided into the following 2 categories: persistent/recurrent depression, and no/transient depression.

The modified Rankin Score (mRS) was used to quantify the functional status at 1-year follow-up. mRS≥2 was regarded as poor prognosis.

### Statistical analyses

Continuous data were presented as the mean (± standard deviation, SD) or medians (interquartile range). Categorical data were reported as frequencies and percentiles. Proportions between groups were compared using the χ^2^ test or Fisher's test. Quantitative data were analyzed by using the *t* test or Wilcoxon rank-sum test. To explore the predictive value of frontal lobe lesions for persistent/recurrent depression, univariate and multivariate logistic regression analyses were used. Gender, NIHSS score and MMSE score at 14±2 days, chronic comorbidities, ischemic lesions on periventricular white matter, frontal lobe and temporal lobe, TOAST classification, taking antidepressants and stroke recurrence within a year after stroke onset were entered in the multivariate logistic regression model by the method of forward LR as confounding variables. To investigate the association between persistent/recurrent depression and poor prognosis, multivariate logistic regression analyses were conducted separately in all patients, the FL group, and the NFL group. Gender, NIHSS score at 14±2 days, and stroke recurrence were entered in the logistic regression models as confounding variables.

For all analyses, a two-tailed probability value of *P*<0.05 was considered statistically significant. All analyses were completed using SPSS statistical software, version 19.0.

## Results

A total of 1,067 patients with first-ever ischemic stroke were included in the analyses. Of these, 376 (35.2%) patients were female. The mean age was 61.5±11.5 years. Compared with the 646 patients withdrawing from the follow-up, included patients had milder neurological impairment, as quantified by NIHSS score at 14±2 days [median (Q1–Q3), 2(1–4) vs 2(1–5), *P* = 0.023], and were more likely to be diagnosed with small-vessel occlusion (25.5% vs 18.1%, *P*<0.001) instead of large-artery atherosclerosis (57.6% vs 66.3%, *P*<0.001) according to the Trial of Org 10172 in Acute Stroke Treatment (TOAST) classification [17]. No other significant differences were found between the two groups. (Table 1)

**Table 1:** Comparison of characteristics between the patients enrolled in the study and those who withdrew from the follow-up.

Variables	Enrolled(n = 1067)	Withdraw(n = 646)	*P*
**Demographic characteristics**			
Age,years,mean±SD	61.5±11.5	61.4±12.0	0.759
Female,n(%)	376(35.2)	204(31.6)	0.121
Education level,n(%)			
High school and above	402(37.7)	270(42.0)	0.082
Married,n(%)	1001(93.8)	590(91.5)	0.067
**Vascular risk factors**			
Current smoker,n(%)	361(33.9)	240(37.3)	0.151
Moderate or heavy drinking,n(%)	149(14.0)	111(17.2)	0.073
Hypertension,n(%)	688(64.5)	416(64.5)	0.580
Diabetes,n(%)	258(24.2)	155(24.0)	0.996
Hyperlipidemia,n(%)	206(19.3)	114(17.7)	0.336
**Clinical characteristics**			
Chronic comorbidities,n(%)	353(33.1)	216(33.4)	0.880
Family history of mental disease,n(%)	6(0.6)	1(0.2)	0.324
NIHSS score at 14±2 days,media(Q1–Q3)	2(1–4)	2(1–5)	0.023
MMSE score,media(Q1–Q3)	28(25–30)	28(24–30)	0.765
TOAST classification, n(%)			
Large-artery atherosclerosis	615(57.6)	428(66.3)	<0.001
Cardioembotism	31(2.9)	24(3.7)	0.357
Small-vessel occlusion	272(25.5)	117(18.1)	<0.001
Other determined etiology	21(2.0)	6(0.9)	0.094
Undetermined etiology	43(4.0)	37(5.7)	0.107

SD, standard deviation; NIHSS, National Institutes of Health Stroke Scale; MMSE, Mini Mental State Examination; TOAST, Trial of Org 10172 in Acute Stroke Treatment.

Of the 1,067 patients included in the analyses, 109 (10.2%) patients had ischemic lesions responsible for the indexed stroke event in the frontal lobe and were included into the FL group, while the others (n = 958) were assigned to the NFL group. Fewer patients in the FL group had a history of hypertension (56.0% vs 65.4%, *P* = 0.017) than in the NFL group. Otherwise, there were no differences between the two groups in terms of demographic characteristics, vascular risk factors, family history of psychiatric disorders and frequency of chronic comorbidities (including chronic disease of cardiovascular, respiratory, digestive, urinary system and tumor). The NIHSS score at 14±2 days was similar [2(1–5) vs 2(1–4), *P* = 0.882] in the two groups. Nevertheless, the MMSE score was significantly lower in the FL group than in the NFL group [27(23–29) vs 28(25–30), *P* = 0.029]. As to the TOAST classification, stroke due to cardioembolism and other determined or undetermined etiology presented more frequently in the FL group than in the NFL group (Table 2).

**Table 2:** Comparison of characteristics between patients with and without frontal lobe lesions.

Variables	All Patients (n = 1067)	Groups		
		FL (n = 109)	NFL(n = 958)	*P*
**Demographic characteristics**				
Age,years,mean±SD	61.5±11.5	59.6±11.5	61.7±11.5	0.056
Female,n(%)	376(35.2)	42(38.5)	334(34.9)	0.460
Education level,n(%)				
High school and above	402(37.7)	46(42.2)	356(37.2)	0.311
Married,n(%)	1001(93.8)	104(95.4)	897(93.6)	0.465
**Vascular risk factors**				
Current smoker,n(%)	361(33.9)	31(28.4)	330(34.6)	0.196
Moderate or heavy drinking,n(%)	149(14.0)	18(16.5)	131(13.7)	0.418
Hypertension,n(%)	688(64.5)	61(56.0)	627(65.4)	0.017
Diabetes,n(%)	258(24.2)	22(20.2)	236(24.6)	0.568
Hyperlipidemia,n(%)	206(19.3)	13(11.9)	193(20.1)	0.101
**Clinical characteristics**				
Chronic comorbidities,(%)	353(33.1)	39(35.8)	314(32.8)	0.528
Family history of mental disease,n(%)	6(0.6)	1(0.9)	6(0.6)	0.054
NIHSS score at 14±2 days,media(Q1–Q3)	2(1–4)	2(1–5)	2(1–4)	0.882
MMSE score,media(Q1–Q3)	28(25–30)	27(23–29)	28(25–30)	0.029
TOAST classification, n(%)				<0.001
Large-artery atherosclerosis	615(57.6)	60(55.0)	555(57.9)	0.563
Cardioembotism	31(2.9)	8(7.3)	23(2.4)	0.004
Small-vessel occlusion	272(25.5)	21(19.3)	251(26.2)	0.115
other determined etiology	21(2.0)	6(5.5)	15(1.6)	0.015
Undetermined etiology	43(4.0)	9(8.3)	34(3.5)	0.035
Stroke recurrence at 1-year, n(%)	70(6.6)	5(4.6)	65(6.8)	0.380

Group FL: patients with frontal lobe lesions; Group NFL: patients without frontal lobe lesions. SD, standard deviation; NIHSS, National Institutes of Health Stroke Scale; MMSE, Mini Mental State Examination; TOAST, Trial of Org 10172 in Acute Stroke Treatment.

**Figure 2:**
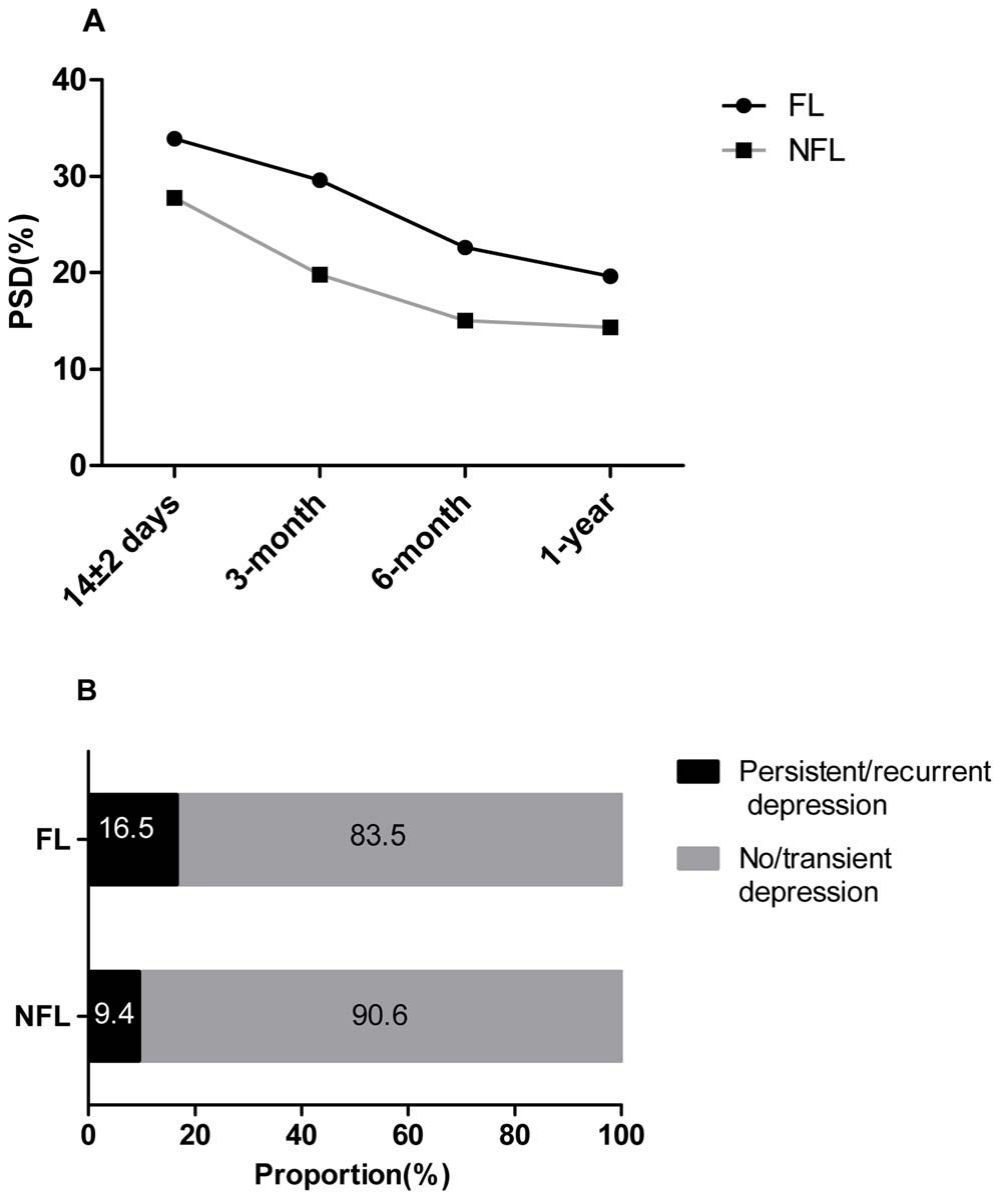
Prevalence of PSD and PSD course categories in the FL and NFL groups. **A.** Prevalence of PSD in the FL and NFL groups at 14±2 days (33.9% vs 27.8%, *p* = 0.175), 3-month (29.6% vs 19.8%, *p* = 0.017), 6-month (22.6% vs 15.0%, *p* = 0.042) and 1-year (19.6% vs 14.3%, *p* = 0.138). **B.** Proportion of two courses of depression categories in the FL and NFL groups, 16.5% vs 9.4%, *p* = 0.028 for persistent/recurrent depression. FL Group, patients with frontal lobe lesions; NFL Group, patients without frontal lobe lesions.

There was a decreasing trend in the incidence of depression during the 1 year follow up, with 303 (28.4%), 220 (20.6%), 166 (15.6)%, and 154(14.4%) cases at 14±2 day, 3 months, 6 months and 1 year, respectively. Figure 2A demonstrates that the FL group had a higher PSD incidence than the NFL group at any of the 4 time points. However, the differences at 14±2 days and 1 year did not reach statistical significance (*P* = 0.175, *P* = 0.138, respectively). Figure 2B shows that the FL group had a significantly higher proportion of patients experiencing persistent/recurrent depression in a year after the stroke onset than the NFL group (16.5% vs 9.4%, *P* = 0.028).

In the univariate analysis, the variables associated with persistent/recurrent depression were female, NIHSS and MMSE scores at 14±2 days, chronic comorbidities, ischemic lesions in periventricular white matter and the frontal lobe, TOAST classification, taking antidepressants and stroke recurrence (all *P*<0.05, Table 3). The multivariate logistic regression analysis showed that after adjusting for variables mentioned above and ischemic lesions in the temporal lobe(*P* = 0.050 in Table 3), ischemic lesions in the frontal lobe was still an independent risk factor for persistent/recurrent depression [odds ratio (OR) 2.025, 95% confidence interval (CI) 1.039–3.949] (Table 4).

**Table 3:** Comparison of characteristics between patients with persistent/recurrent depression and patients with no/transient depression.

Variables	Patients with persistent/recurrent depression (n = 108)	Patients with no/transient depression (n = 959)	*P*
Age(years)	61.8±11.8	61.4±11.5	0.757
Female,n(%)	50(46.3)	326(34.0)	0.011
Education level,n(%)			
High school and above	36(33.3)	366(38.2)	0.318
Married,n(%)	99(91.7)	902(94.1)	0.328
Current smoker,n(%)	38(35.2)	323(33.9)	0.782
Moderate and heavy drinking,n(%)	15(13.9)	134(14.0)	0.981
Family history of psychiatric disorders,n(%)	1(0.9)	5(0.5)	0.796
Hypertension,n(%)	71(65.7)	617(64.3)	0.898
Diabetes,n(%)	29(26.9)	229(23.9)	0.795
Hyperlipidemia,n(%)	27(25.0)	179(18.7)	0.284
Chronic comorbidities,n(%)	45(41.7)	308(32.1)	0.046
NIHSS score at 14±2 days,media(Q1–Q3)	4(2–7)	2(1–4)	<0.001
MMSE score,media(Q1–Q3)	26(22–28)	28(25–30)	<0.001
Lesion location,n(%)			
Frontal lobe	18(16.7)	91(9.5)	0.020
Temporal lobe	17(15.7)	93(9.7)	0.050
Parietal-occipital lobe	21(19.4)	149(15.5)	0.293
Periventricular white matter	7(6.5)	24(2.5)	0.042
Basal ganglia	56(51.9)	495(51.6)	0.963
Infratentorial region	27(25.0)	256(26.7)	0.705
TOAST classification, n(%)			0.002
Large-artery atherosclerosis	66(61.1)	549(57.2)	0.441
Cardioembotism	8(7.4)	23(2.4)	0.003
Small-vessel occlusion	15(13.9)	257(26.8)	0.004
Other determined or undertermined	8(7.4)	54(5.6)	0.454
Taking antidepressant,n(%)			
14±2 days	13(12.0)	55(5.7)	0.011
3-month	16(14.8)	36(3.8)	<0.001
6-month	16(14.8)	35(3.7)	<0.001
1-year	15(13.9)	27(2.9)	<0.001
Stroke recurrence at 1-year,n(%)	17(15.7)	53(5.5)	<0.001

SD, standard deviation; NIHSS,National Institutes of Health Stroke Scale; MMSE,Mini Mental State Examination; TOAST,Trial of Org 10172 in Acute Stroke Treatment.

**Table 4:** Association between frontal lobe lesions and time course of depression in patients with first-ever ischemic stroke (persistent/recurrent depression versus no/transient depression).

Variable	Unadjusted OR			Adjusted OR*		
	*OR*	*95%CI*	*P*	*OR*	*95%CI*	*P*
Frontal lobe lesions	1.908	1.100–3.307	0.021	2.025	1.039–3.949	0.038

*Adjusted for gender, NIHSS score and MMSE score at 14±2 days, chronic comorbidities, ischemic lesions on periventricular white matter or temporal lobe, TOAST classification, antidepressants treatment and stroke recurrence within a year.

OR, odds ratio; CI, confidential interval.

**Figure 3:**
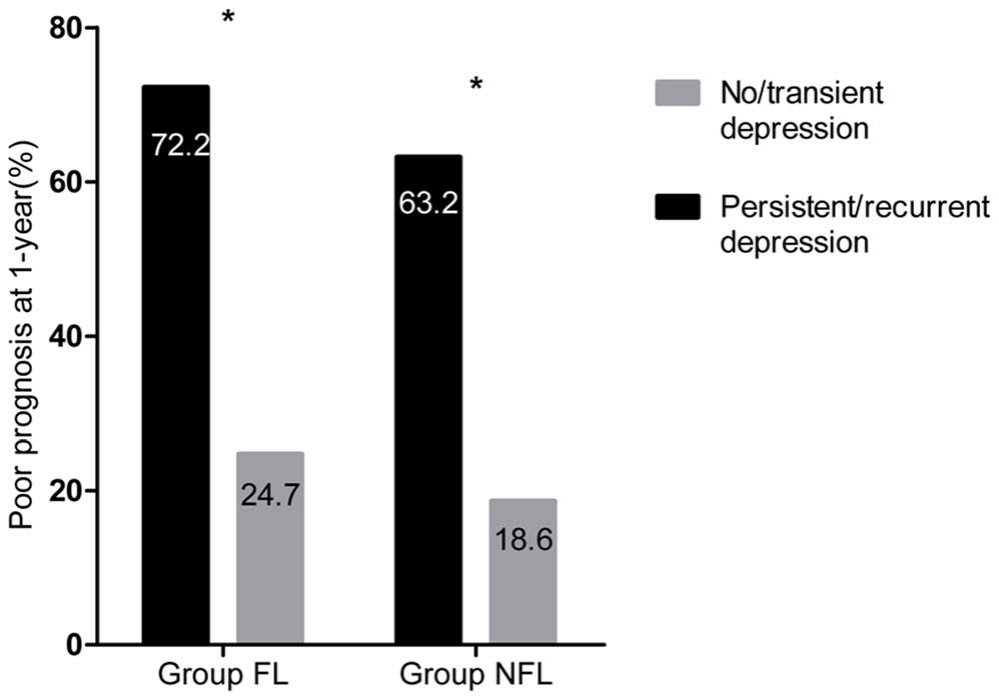
1-year prognosis of patients in the FL and NFL groups. Comparison of the 1-year proportion of poor prognosis (mRS≥2) between two course of PSD categories in the FL and NFL groups after first-ever ischemic stroke. *p<0.001. FL Group, patients with frontal lobe lesions; NFL Group, patients without frontal lobe lesions.

More patients in the FL group had poor prognosis (mRS≥2) at 1-year than in the NFL group (32.7% vs. 22.7%, *P* = 0.021). The patients with persistent/recurrent depression had significantly worse functional outcome than patients with no/transient depression in both the FL and NFL groups (72.2% vs 24.7%, *P*<0.001 in the FL group; 63.2% vs 18.6%, *P*<0.001 in the NFL group, respectively) (Figure 3).

After adjusting for gender, NIHSS score at 14±2 days and stroke recurrence, compared with no/transient depression, persistent/recurrent depression was significantly associated with poor prognosis at 1-year in all patients (OR 4.868, 95% CI 2.949–8.037), the FL group (OR 5.981, 95% CI 1.680–21.291) and the NFL group (OR 4.445, 95% CI 2.568–7.695).

## Discussion

To the best of our knowledge, this was the first longitudinal study to investigate the relationship between frontal lobe infarction and the course of depression in patients with first-ever ischemic stroke. Patients with frontal lobe lesions were at a higher risk for persistent or recurrent depression and poor outcome at 1 year after stroke compared to patients with lesions located elsewhere. Patients with persistent or recurrent depression had worse outcomes than those with no or transient depression irrespective of the presence of frontal lobe lesions.

### Frontal lobe lesions and PSD

There have been several attempts to establish a relationship between the localization of stroke and the occurrence of PSD, but the results from the existing studies remain contradictory. A systematic review offered no support for the hypothesis that the risk of PSD was affected by the hemispheric location of brain lesion or by the position of the lesion along the antero-posterior axis [18]. Nevertheless, after the publication of the systematic review mentioned above, several studies have reported that frontal lobe lesions are associated with PSD in patients with stroke. The inconsistency of findings is partly related to the fact that neuroimaging methods, unlike neuropathology, can not precisely assess the full extent of stroke-affected areas or specify the different types of vascular lesions [19].

Neuroimaging markers of cellular function, functional and structural abnormalities have been found in the prefrontal and limbic structures in major depressive disorder [20]–[22]. The following three neural circuits are implicated in the pathophysiology of major depressive disorder: the limbic-cortical-striatal-pallidal-thalamic (LCSPT) circuits, the orbital prefrontal network and medial prefrontal networks [23]. It is postulated that these neural circuits are also involved in the pathophysiology of PSD. Plenty of studies conducted recently have provided some evidence for this postulation. Terroni et al. suggested that PSD was etiologically related to the disruption of the left LCSPT circuits [12]. Lesions affecting structures of the prefrontal-subcortical circuit, particularly in the left hemisphere have been reported to predispose stroke patients to depression [8], [24]. Infarcts in the frontal subcortical circuits (FSC) were found to be independent predictors for PSD in patients with ischemic stroke [13]. A MRI spectroscopy study also found that PSD was accompanied by metabolic changes in the frontal lobe [25].

There may be different biological or psychological processes involved in PSD between the chronic phase and the subacute or acute phases of ischemic stroke. The neural circuits mentioned above could be disrupted by the secondary degeneration besides primary ischemic lesion, due to retrograde degeneration, anterograde degeneration and/or extensive vasogenic edema [26]. The aging brain seems to be more sensitive to the mechanisms of secondary degeneration [27]. Neurotransmitter synthesis could also be affected by secondary neurodegeneration [28].

The present study also found that frontal lobe lesions were associated with persistent or recurrent depression in the longitudinal assessment. We hypothesize that the combination of primary ischemic lesions, secondary degeneration and the long-term adverse psychological effects related to the stroke event and functional impairment are etiological factors in persistent/recurrent PSD.

### PSD and functional outcomes

Findings regarding the association between PSD and functional outcomes are inconsistent and depression has been found to be related [29], [30] and unrelated to [31], [32] functional outcomes after stroke. The time of examination of the relationship between depression and functional recovery may be one reason for the inconsistency. Persistent depression and recurrent depression were found to be the top 2 independent determinants of 1-year outcome after stroke in our previous study, with an OR (95% CI) of 7.615 (5.011–11.572), and 4.701 (2.721–8.122), respectively [4]. Here, we found that the risk of functional impairment (mRS≥2) at 1-year in patients with persistent or recurrent depression was approximately 5 times that of patients with transient depression or without mood disorder. In the FL group, approximately 75% of patients with persistent/recurrent depression were functionally impaired at 1-year (mRS≥2). Compared with patients without depression or with transient depression, the risk of poor prognosis at 1-year was 6 and 4 times that of patients with persistent or recurrent depression in the FL group and NFL group, respectively.

Previous studies have shown that patients with PSD benefited from antidepressant therapy [33], [34]. Antidepressants also showed promising results for preventing PSD and recovery from disability independent of the presence of depression [35], [36]. However, the report by Schmid et al. regarding the relationship between the improvement of depression and functional recovery at 12 weeks was inconclusive [37]. In our study, 22.4%, 23.6%, 30.7%, and 27.3% of PSD patients received antidepressants at 14±2 days, 3 months, 6 months and 1 year visits, respectively (not listed in the table), similar to the findings from a Swedish study (22.5% of men and 28.1% of women at 3 months) [38]. In the Secondary Prevention of Small Subcortical Stroke (SPS3) study, 19% patients who were classified as depressed at all annual visits over a 4 year period were on antidepressants [39]. Less than 15% of patients with persistent or recurrent PSD accepted antidepressants at any time point of the follow up in the present study. The lack of antidepressant treatment might also contribute to the persistent or recurrent course of PSD.

### Limitations

The study has a number of limitations. First, patients with severe language or cognitive impairments were excluded. Second, inter-rater reliability tests were not performed for the multicenter assessment of CT or MRI scans. Furthermore, depression intervention only focused on antidepressants, while psychological interventions and social support were not measured in the treatment of PSD, which might affect the course of depression. Finally, patients enrolled in the study had mild neurological functional impairment (median of NIHSS score was 2), which might lead to an underestimation of the incidence of PSD. PSD may be more common in patients with severe functional impairments [40].

## Conclusions

Despite these limitations, the study findings have some important implications. One in 6 patients with frontal lobe lesions had persistent or recurrent PSD in the first year after stroke onset, which was twice as many as patients without frontal lobe lesions. Frontal lobe lesions were significantly associated with a persistent or recurrent course of PSD in patients with first-ever ischemic stroke. Patients with frontal lobe lesions had poorer functional status at 1-year compared with patients without detectable frontal lobe damage. Approximately 75% of patients with frontal lobe lesions and persistent/recurrent PSD had poor functional outcome at 1-year.It is critical that all stroke survivors are periodically screened for depression and provided with appropriate treatments and follow-ups to ensure the adequacy of the treatments, especially in patients with frontal lobe lesions due to the higher risk for a persistent or recurrent course of PSD and poor functional outcomes.
